# Obstructive jaundice due to von Hippel-Lindau disease-associated pancreatic lesions: A case report

**DOI:** 10.3892/ol.2014.2098

**Published:** 2014-04-28

**Authors:** XIAOYU LIANG, FANGUO HU, ZHICHENG MA, NAN LI, YAN CHEN, JIE ZHANG

**Affiliations:** 1 Department of General Surgery, General Hospital of Tianjin Medical University, Tianjin 300052, P.R. China; 2 Department of General Surgery, Tianjin First Center Hospital, Tianjin 300192, P.R. China

**Keywords:** von Hippel-Lindau disease, pancreatic neoplasm, obstructive jaundice

## Abstract

von Hippel-Lindau (VHL) disease is an autosomal dominantly inherited neoplastic syndrome that may lead to pancreatic masses and obstructive jaundice. The present study describes the case of a 20-year-old male who suffered from obstructive jaundice due to VHL disease-associated pancreatic lesions whose primary symptom was dizziness, followed by the appearance of jaundice. Since the excision of the renal cell carcinomas was not possible, the patient also refused surgery to resect the pancreatic head mass. A metallic stent was placed at the stenosis site of the common bile duct. Percutaneous transhepatic cholangiography (PTCD) surgery was later performed following complete blockage of the stent, however, to date, the patient continues to rely on PTCD. VHL disease-associated pancreatic lesions are rarely the direct cause of mortality, however, obstructive jaundice due to these lesions may be lethal. Therefore, the treatment of patients with incurable renal or central nervous system tumors and obstructive jaundice presents a problem.

## Introduction

von Hippel-Lindau (VHL) disease is an autosomal dominantly inherited neoplastic syndrome that is characterized by hemangioblastomas of the central nervous system (CNS) and retina, renal cell carcinoma (RCC), pheochromocytoma, pancreatic neuroendocrine tumors (NETs) and endolymphatic sac tumors (1,2). The majority of patients are diagnosed following the identification of CNS tumors. The incidence of VHL disease is ~1 in 36,000 live births and its penetrance is >90% by the age of 65 years (3). Tumors of the CNS and RCCs are the main causes of mortality in VHL patients, accounting for the median life expectancy of <50 years (4,5). Well-defined diagnostic criteria have been identified for VHL disease. If there is a family history, a diagnosis of VHL disease can be determined by identifying only a single VHL tumor, whereas if there is no family history, the presence of two VHL tumors is required for diagnosis (6). VHL disease is classified into types 1 and 2 according to the absence or presence of pheochromocytoma, respectively. Type 2 is further divided into types 2A, 2B and 2C. Type 2A is characterized by a high risk of hemangioblastoma and pheochromocytoma, with a lower risk of RCC, and type 2B is characterized by a high risk of pheochromocytoma, RCC and pheochromocytoma. Cases that present with only pheochromocytoma are designated as type 2C (6,7).

The VHL gene is localized on the short arm of chromosome 3, with three exons, and coded for two isoforms of pVHL (a tumor suppressor protein of VHL). pVHL predominantly acts by the direct regulation of the levels of hypoxia-inducible transcription factor (HIF) types 1 and 2, which are significant in the cellular response to oxygen deficiency. When VHL gene mutations (mostly deletions) occur and pVHL is not produced, HIF is consequently not blocked, resulting in the promotion of angioneogenesis and uncontrolled cell proliferation. Thus, VHL gene mutations significantly increase the risk of tumor growth in target organs (8,9). The present study describes the case of a 20-year-old male who suffered from obstructive jaundice due to VHL disease-associated pancreatic lesions. The patient provided written informed consent for the publication of this study.

## Case report

**Figure 1:**
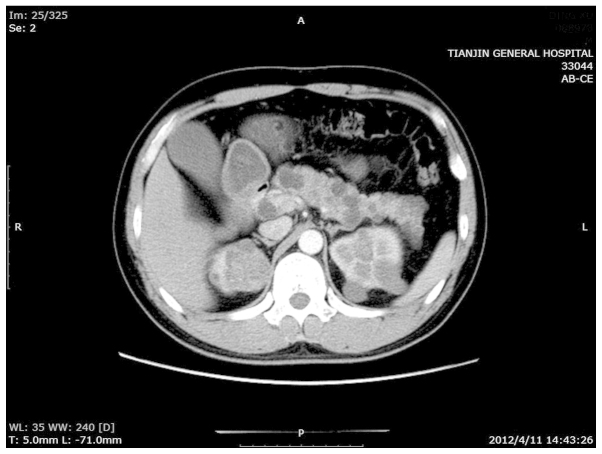
Enhanced computed tomography scan showing the lesion in the pancreas and kidneys.

**Figure 2:**
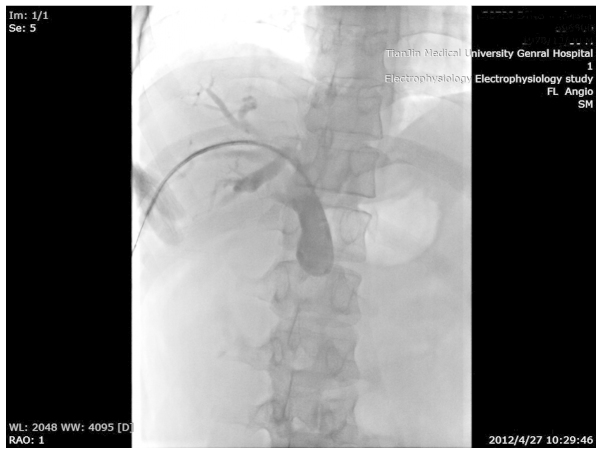
Cholangiography showing the cholangiectasis.

**Figure 3:**
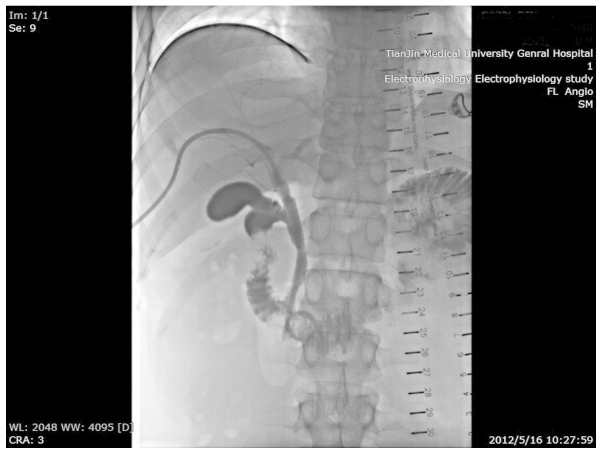
Cholangiography showing the metallic stent placed at the stenosis site of the common bile duct.

In 1998, a 20-year-old male was admitted to the General Hospital of Tianjin Medical University (Tianjin, China) with symptoms of dizziness, unsteadiness and nausea for three weeks, as well as vomiting for one week. Magnetic resonance imaging (MRI) revealed a mass in the cerebellar vermis and surgery was performed to excise the tumor. The pathological examination identified a hemangioblastoma. Three years later, the patient was readmitted to hospital due to dizziness lasting for one week. An MRI examination showed recurrence of the hemangioblastoma in the cerebellum and the patient underwent surgery to resect the tumor. In April 2012, the patient was readmitted to hospital for the third time due to jaundice. An enhanced computed tomography (CT) scan of the abdomen revealed multiple RCCs in the kidneys, and a nodule with a rich blood supply in the pancreatic head. In addition, numerous cysts were identified throughout the pancreas (Fig. 1). These observations were confirmed by magnetic resonance cholangiopancreatography, which revealed that the nodule in the pancreatic head was ~2.9×2.2 cm in size and possibly a NET. Due to the size and location of the tumor, the bile duct in the pancreas was compressed and the upper parts of the common bile and hepatic ducts were dilated. Since it was not possible to excise the RCCs, the patient also refused surgery to resect the pancreatic head mass. A metallic stent was placed at the stenosis site of the common bile duct, which alleviated the jaundice (Figs. 2 and 3). Nine months later, the patient returned to the hospital with a fever, abdominal pain and jaundice. An enhanced abdominal CT was performed, which revealed no change in the size of the pancreatic head mass. The patient’s symptoms were relieved following anti-inflammatory therapy for one week. However, the patient continued to suffer the same symptoms every two months, and gradually, anti-inflammatory therapy failed to alleviate the symptoms. Radiography tests revealed complete blockage of the stent and thus, percutaneous transhepatic cholangiography (PTCD) surgery was performed.

Genomic DNA was extracted from the peripheral blood leukocytes and polymerase chain reaction was performed. Direct sequencing revealed a known mutation of a base pair change at nucleotide 473 in exon 3 (T473T/C) of the VHL gene, resulting in the amino acid change Leu158Pro.

There was a recorded family history of VHL disease, with the patient’s mother, grandmother, two uncles and three aunts also suffering from the disease. Additionally, one uncle had succumbed to RCC and five other individuals in the family had succumbed to cerebral hemangioblastomas.

## Discussion

VHL-associated pancreatic lesions are usually non-functional and asymptomatic. A number of different pancreatic lesions have been associated with VHL disease, including true cysts, serous cystadenomas and NETs. In the majority of cases, VHL-associated pancreatic NETs are also non-functional, asymptomatic and typically slow growing (10). Charlesworth *et al* (11) reviewed 11 studies (excluding case studies) of VHL-associated pancreatic lesions and identified that 211 (15%) of the 1,442 patients with VHL also exhibited pancreatic NETs. Furthermore, metastasis was observed in 27 VHL patients (12.8%) of the 211 patients with concurrent pancreatic NET, whereas in patients with primary pancreatic NET, distant metastases were reported at diagnosis in 64% of patients. Libutti *et al* (12) reported that the median size of lesions in the pancreas of patients exhibiting metastatic disease was 5 cm, whereas in patients without metastatic disease, the median size was 2 cm. In the present study, the pancreatic NET was also non-functional, and no marked change in size was identified after one year. However, since the tumor was located at the head of pancreas, the bile duct in the pancreas was compressed and the upper parts of the common bile and hepatic ducts were dilated. As a result, the patient suffered from severe jaundice and abdominal pain. Although VHL-associated pancreatic NETs may be asymptomatic, they may cause certain obstructive symptoms by blocking the common bile and pancreatic ducts.

In patients without VHL, pancreatic NETs should be treated according to the degree of differentiation and malignancy. Surgical resection may be recommended following the evaluation of the functionality, disease stage and metastasis of the tumor (13). However, in patients with VHL-associated pancreatic NET, surgical treatment must be selected carefully as this type of tumor is rarely the direct cause of mortality (14). Blansfield *et al* (14) reported that pancreatic NETs were the cause of mortality in only 0.3% of patients with VHL (633 patients in total) and in 1.9% of patients exhibiting concurrent pancreatic NET and VHL (108 patients). In the majority of cases, the cause of mortality in patients with VHL is CNS hemangioblastoma or renal cancer. Accordingly, the prognosis of pancreatic NET associated with VHL is considered to be favorable. Therefore, in patients with VHL, the pancreatic NETs must be treated based on tumor size. Libutti *et al* (12,15) recommended surgery when the tumor size is ≥3 cm in the pancreatic tail region and ≥2 cm in the pancreatic head region. Furthermore, Blansfield *et al* (14) recommended surgery when the tumor size is ≥3 cm. The studies also proposed a tumor doubling time of <500 days as an additional factor to be considered when selecting surgical treatment.

As the tumor in the present case was located at the head of the pancreas and was >2 cm in size, according to the standard reported by Libutti *et al* (15) surgery to resect the tumor was the recommended treatment. However, the patient also suffered from unresectable bilateral RCC, so whether it is reasonable to perform a procedure, such as Whipple surgery, in such a case requires further discussion. Based on the evidence that the RCC was incurable, the patient refused Whipple surgery. The manner in which to deal with such a case also requires investigation, as to date, no studies have analyzed this problem. For the current patient, a metallic stent was placed at the stenosis site of the common bile duct, however, complete blockage of the stent was revealed nine months later. PTCD was performed, but it is expected that the effects of the surgery will not last long.

## References

[1] Richard S, Graff J, Lindau J, Resche F (2004). Von Hippel-Lindau disease. Lancet.

[2] Molino D, Sepe J, Anastasio P, De Santo NG (2006). The history of von Hippel-Lindau disease. J Nephrol.

[3] Kim JJ, Rini BI, Hansel DE (2010). Von Hippel Lindau syndrome. Adv Exp Med Biol.

[4] Lonser RR, Glenn GM, Walther M (2003). von Hippel-Lindau disease. Lancet.

[5] Tootee A, Hasani-Ranjbar S (2012). Von hippel-lindau disease: a new approach to an old problem. Int J Endocrinol Metab.

[6] Maher ER, Neumann HP, Richard S (2011). Von Hippel-Lindau disease: a clinical and scientific review. Eur J Hum Genet.

[7] Zbar B, Kishida T, Chen F (1996). Germline mutations in the Von Hippel-Lindau disease (VHL) gene in families from North America, Europe, and Japan. Hum Mutat.

[8] Clark PE, Cookson MS (2008). The von Hippel-Lindau gene: turning discovery into therapy. Cancer.

[9] Baldewijns MM, van Vlodrop IJ, Vermeulen PB (2010). VHL and HIF signalling in renal cell carcinogenesis. J Pathol.

[10] Tamura K, Nishimori I, Ito T, Yamasaki I, Igarashi H, Shuin T (2010). Diagnosis and management of pancreatic neuroendocrine tumor in von Hippel-Lindau disease. World J Gastroenterol.

[11] Charlesworth M, Verbeke CS, Falk GA (2012). Pancreatic lesions in von Hippel-Lindau disease? A systemic review and meta-synthesis of the literature. J Gastrointest Surg.

[12] Libutti SK, Choyke PL, Alexander HR (2000). Clinical and genetic analysis of patients with pancreatic neuroendocrine tumors associated with von Hippel-Lindau disease. Surgery.

[13] NCCN Clinical Practice Guidelines in Oncology (NCCN Guidelines™) (2011). Neuroendocrine tumors.

[14] Blansfield JA, Choyke L, Morita SY, Choyke PL, Pingpank JF, Alexander HR (2007). Clinical, genetic and radiographic analysis of 108 patients with von Hippel-Lindau disease (VHL) manifested by pancreatic neuroendocrine neoplasms (PNETs). Surgery.

[15] Libutti SK, Choyke PL, Bartlett DL, Vargas H, Walther M, Lubensky I (1998). Pancreatic neuroendocrine tumors associated with von Hippel Lindau disease: diagnostic and management recommendations. Surgery.

